# Effectiveness and safety of brivaracetam in comparison with levetiracetam in seizures

**DOI:** 10.1186/s42494-025-00229-z

**Published:** 2025-10-02

**Authors:** Shalini Sivadasan, Flencinecia Basil Raj, Kevin John, Sowmya Murugan, Stephy Susan Sam, Senthil Kumar Elumalai

**Affiliations:** 1 Department of Pharmacy Practice, KMCH College of Pharmacy, Coimbatore, Tamil Nadu 641035 India; 2 Consultant Neurologist, Kovai Medical Center and Hospital, Avinashi Road, Coimbatore, Tamil Nadu 641048 India

**Keywords:** Effectiveness, Levetiracetam, Brivaracetam

## Abstract

**Background:**

There are increasing incidence of psychiatric side effects associated with the use of anti-epileptics. Prospective observational studies on the effectiveness and safety of levetiracetam (LEV) and brivaracetam (BRV), along with the haematological abnormalities of both treatments, in seizure patients in an Indian population are lacking. Therefore, we aimed to compare the effectiveness and safety of LEV and BRV in seizure patients and evaluated behavioural and non-behavioural side effects, as well as outcomes when switching between LEV and BRV.

**Methods:**

A prospective observational study was conducted in newly diagnosed as well as previously diagnosed patients (*n* = 115) with epilepsy aged ≥ 5 years of age receiving LEV (*n* = 66) or BRV (*n* = 49). Baseline data were collected during the initiation of the study and were compared to the data obtained at the end of the study. A seizure severity questionnaire was used to assess the severity of seizures, and a brief psychiatric rating scale, Hamilton anxiety rating scale, and pediatric epilepsy side effects questionnaire were used to assess the behavioural and non-behavioural side effects.

**Results:**

At baseline, adults taking LEV showed higher rates of behavioral adverse events (BAEs) compared to those on BRV. During follow-up, the most common behavioural adverse event reported in both treatment groups (LEV and BRV) was depression. The most frequently reported non-behavioural side effect in patients taking BRV was drowsiness. Patients who switched from LEV to BRV due to psychiatric side effects showed positive results with BRV (*n* = 5).

**Conclusions:**

In summary, the study found that BRV is a safe alternative, with fewer and less severe side effects compared to LEV. While LEV showed slightly higher efficacy and a lower probability of drowsiness, BRV proved more tolerable for patients experiencing LEV-induced side effects. Switching from LEV to BRV decreased the psychiatric side effects.

**Supplementary Information:**

The online version contains supplementary material available at 10.1186/s42494-025-00229-z.

## Background

Epilepsy is a neurological disorder characterized by two or more unprovoked seizures occurring more than twenty-four hours apart [1]. A seizure is a result of abnormal, excessive synchronous neuronal activity in the brain [2]. Epilepsy is one of the most prevalent neurological disorders in India, where it affects an estimated 12 million people and accounts for around one-sixth of the global burden [3]. Psychiatric and behavioural disorders are more common in people with epilepsy, possibly due to anti-seizure medications (ASMs) or the disease itself [4, 5]. Despite these side effects, pyrrolidone derivatives, such as levetiracetam **(**LEV) and brivaracetam (BRV), offer effective treatment. There are very few prospective observational studies that compare LEV and BRV in seizures, changes in side effects when switching from LEV to BRV or BRV to LEV, as well as comparisons between BRV and LEV in terms of hematological parameters in Indian populations, highlighting the importance of accurate comparative data. Although BRV and LEV bind to the synaptic vesicle 2A (SV2A) protein at structurally similar locations, they interact with different binding sites or conformations [6]. BRV has a 15–30-fold higher binding affinity and more selective interaction with the SV2A protein than LEV does [7]. Despite their efficacy, LEV and BRV may cause adverse behavioural or psychiatric events [8]. Several case reports emphasize the behavioural adverse events (BAEs) associated with LEV in the Indian population [9–12]. Therefore, in this study, our objectives were to assess the effectiveness and safety of BRV and LEV in patients with epilepsy, evaluate hematological parameter changes with BRV or LEV treatment, and monitor behavioural side effect changes during switching from LEV to BRV or BRV to LEV.

## Methods

### Study design

This was a prospective observational study conducted at a tertiary care hospital, Coimbatore, Tamil Nadu, India. Seizure severity and psychiatric assessments of adults and pediatric patients were performed on the basis of the scales and questionnaires at baseline and three subsequent reviews. Hematological abnormalities were also assessed by reviewing the patients’ laboratory data.

### Study setting and participant characteristics

This study was approved by the Institutional Human Ethics and Scientific Committee (EC/AP/1037/04/2023)**.** Outpatients and inpatients were recruited from the department of neurology from April 2023 to September 2023. The study included both inpatients and outpatients above the age of 5 years diagnosed with any type of seizure taking BRV or LEV; newly diagnosed epileptic patients who were prescribed with LEV or BRV. Those patients with major psychiatric pathologies before the initiation of LEV or BRV; cancer patients; and pregnant women were excluded from the study.

### Description of materials

Pediatric and adult patients were assessed for seizure severity via the Seizure Severity Questionnaire (SSQ), and psychiatric assessment was performed via the Brief Psychiatric Rating Scale (BPRS) for adults and the Pediatrics Epilepsy Side Effects Questionnaire (PESQ) for pediatrics and the Hamilton Anxiety Rating Scale (HAM-A) for both adults and pediatrics.

#### Seizure severity questionnaire (SSQ)

This 24-item SSQ was used to assess alterations in seizure intensity and bothersomeness. It is divided into three sections: the first section assesses the patient before the seizure, consisting of one question; the next section contains two questions evaluating the patient during the seizure; and the last section has seven questions, assessing the patient post-seizure, including severity and bothersomeness. Each question uses a 7-point scale (1 = never to 7 = always; 1 = no bother at all to 7 = very bothersome), with higher scores denoting greater severity.

#### Brief psychiatric rating scale (BPRS)

The BPRS is an 18-item, clinician-based rating scale that highlights notable psychiatric symptoms. It uses a seven-point Likert scale, ranging from "not present" to "extremely severe", to determine symptom severity. The BPRS is in the public domain.

#### Pediatric epilepsy side effects questionnaire (PESQ)

This 19-item PESQ was used in pediatric populations to evaluate adverse effects of ASMs. It has five subscales: cognitive, motor, behavioural, general neurological, and weight. The scores of the items ranged from 1 ("not present/not applicable or unable to assess") to 6 ("high severity"). It is a valid and reliable tool for assessing side effects across various epilepsy diagnoses.

#### Hamilton anxiety rating scale (HAM-A)

This 14-item HAM-A evaluates anxiety symptoms and is commonly used as an outcome measure in the treatment of generalized anxiety disorder. It is available in the public domain. Each item has a score ranging from 0 ("not present") to 4 ("very severe").

#### Description of materials

**Fig. 1:**
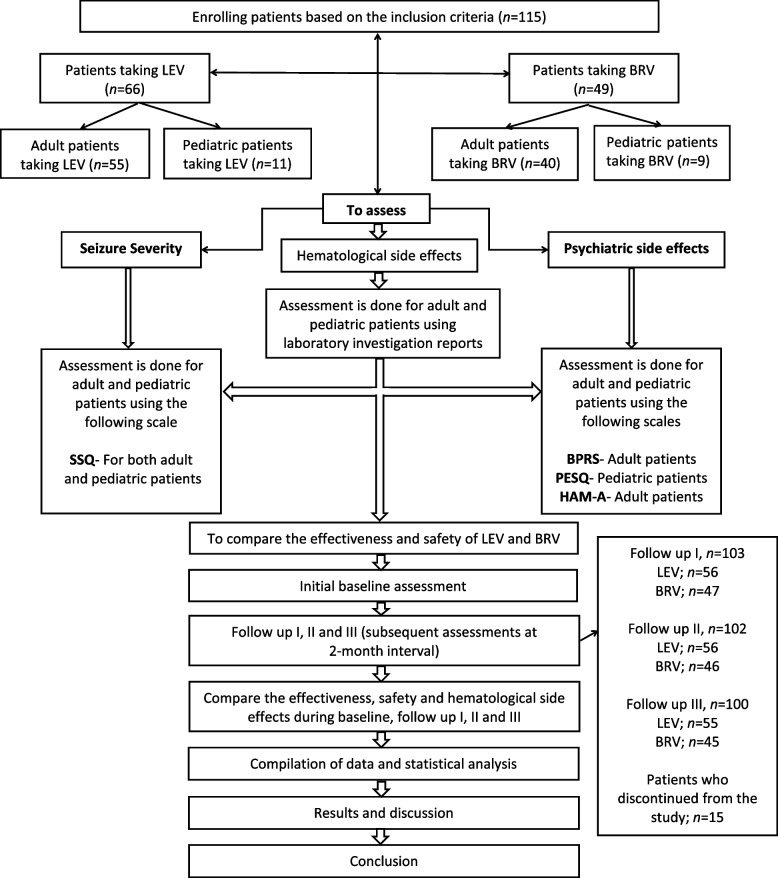
Flow chart of the entire study process

The data collection form was designed to collect information such as patient demographics, illness history, seizure frequency, hematological laboratory parameters, and medical/medication history from the patients, with documented occurring at the beginning of the study. Seizure severity and psychiatric assessments were carried out via appropriate assessment scales or questionnaires mentioned before. Baseline data were collected at the time of inclusion for both newly diagnosed and previously diagnosed epilepsy patients (limited to those receiving LEV or BRV monotherapy). Patients receiving BRV were prescribed various dosage forms, including tablets (25 mg, 50 mg, 75 mg, 100 mg, 150 mg, 175 mg, and 200 mg) and syrup (2 mL and 5 mL). Similarly, patients on LEV were administered tablets (75 mg, 500 mg, 750 mg, 1000 mg, 1500 mg, and 2000 mg) and syrup (4 mL, 8 mL, and 10 mL). Patients were reviewed for three subsequent follow-ups (Fig. 1), each review conducted at every two months intervals. At each follow-up, the patients were assessed for seizure severity and side effects of LEV and BRV via scales and questionnaires. Patients were excluded due to poor adherence, including treatment non-compliance, lack of cooperation with the study procedures, or voluntary discontinuation.

#### Statistical analysis

All collected data were organized and entered into Microsoft Excel. The statistical analysis was performed using SPSS version 26. Clinically significant changes in the SSQ, BPRS, HAM-A, and PESQ scores were assessed through the mean change in scores and standard deviation. The chi-square test was utilized in this study to compare the two treatment arms for safety and effectiveness. Hematological abnormalities were compared between groups using frequency and percentage analyses. Pearson’s chi-square test and Fischer’s exact test were used to determine statistical significance.

#### Sample size

Sample size was calculated according to Snoeren et al. [13], and the minimum sample size required in each group to conduct the study is 45 (Total sample size = 90).

## Results

Among 115 patients of South Indian origin enrolled in the study from April 2023 to September 2023, 95 (82.6%) were adults (aged ≥ 18 years) and 20 (17.3%) were pediatric patients (aged 5–17 years). Among the adult patients, 55 (57.8%) received LEV and 40 (42.1%) received BRV. Among the pediatric patients, 11 (55%) were on LEV and 9 (45%) were on BRV. Individuals aged 18-40 years predominated in both groups (36.4% in LEV; 32.7% in BRV). Males accounted for 62% (*n* = 71) of the patients versus 38% (*n* = 44) females. Overall, 66 (57.3%) patients were on LEV and 49 (42.6%) patients were on BRV at baseline (Table 1). Treatment effectiveness was analyzed by responder rates (≥ 50% reduction in seizure frequency) in the two treatment groups at three subsequent follow-up . At each follow-up, patients were identified as either positive responders (seizure-free) or negative responders to LEV and BRV (Tables 2). The mean score distribution of all assessment scales is shown in Table 3.

**Table 1:** Gender and age distribution among patients taking LEV and BRV

Category	LEV (*n* = 66)	BRV (*n* = 49)
**Gender distribution**		
Female (*n* = 44)	26 (59.0%)	18 (41.0%)
Male (*n* = 71)	40 (56.3%)	31 (43.7%)
**Age distribution (years)**		
*** Adult (n* = *95)***	***55 (58.0%)***	***40 (42.0%)***
18–40 (*n* = 40)	**24 (60.0%)**	**16 (40.0%)**
41–64 (*n* = 36)	22 (61.0%)	14 (39.0%)
> 65 (*n* = 19)	9 (47.4%)	10 (52.6%)
*** Pediatric (n* = *20)***	***11 (55.0%)***	***9 (45.0%)***
5–11 (*n* = 12)	8 (66.7)	4 (33.3%)
12–17 (*n* = 8)	3 (37.5%)	5 (62.5%)

*LEV* Levetiracetam, *BRV* Brivaracetam

**Table 2:** Positive and negative responders of the treatment in each follow-up

Follow-up period	Response category	Patients, *n* (%)
First follow-up (*n* = 103)	Positive responders	94 (91.2%)
	Negative responders	9 (8.7%)
	LEV patients	3 (33.3%)
	BRV patients	6 (66.6%)
Second follow-up (*n* = 102)	Positive responders	100 (98%)
	Negative responders	2 (1.9%)
	LEV patients	1 (1.9%)
	BRV patients	1 (1.9%)
Third follow-up (*n* = 100)	Positive responders	99 (99%)
	Negative responders	1 (1%)
	LEV patients	0
	BRV patients	1 (1%)

*BRV* Brivaracetam, *LEV* Levetiractam

**Table 3:** Mean score distribution of all the scales used in the study

BASELINE				
**Scale**	**Score**	**LEV (*n* = 66)**	**BRV (*n* = 49)**	***P*-value**
** SSQ**	0–0.47 (*n* = 54)	39 (59.1%)	15 (30.6%)	0.002
	≥ 0.48 (*n* = 61)	27 (40.9%)	34 (69.4%)	
** HAM-A**	0–7 (*n* = 44)	26 (39.4%)	18 (36.7%)	0.673
	8–14 (*n* = 42)	26 (39.4%)	16 (32.7%)	
	15–23 (*n* = 22)	11 (16.7%)	11 (22.4%)	
	≥ 24 (*n* = 7)	3 (4.5%)	4 (8.2%)	
** BPRS**	0–31 (*n* = 57)	33 (60.0%)	24 (60.0%)	0.754
	32–41 (*n* = 23)	15 (27.3%)	8 (20.0%)	
	42–53 (*n* = 6)	3 (5.5%)	3 (7.5%)	
	≥ 54 (*n* = 9)	4 (7.3%)	5 (12.5%)	
***Follow-up I***				
** SSQ**	0–0.47 (*n* = 2)	1 (1.8%)	1(2.1%)	0.9
	≥ 0.48 (*n* = 101)	55 (98.2%)	46 (97.9%)	
** HAM-A**	0–7 (*n* = 68)	35 (62.5%)	33 (70.2%)	0.649
	8–14 (*n* = 25)	16 (28.6%)	9 (19.1%)	
	15–23 (*n* = 7)	4 (7.1%)	3 (6.4%)	
	≥ 24 (*n* = 3)	1 (1.8%)	2 (4.3%)	
** BPRS**	0–31 (*n* = 71)	37 (82.2%)	34 (87.2%)	0.396
	32–41 (*n* = 8)	6 (13.3%)	2 (5.1%)	
	42–53 (*n* = 5)	2 (4.4%)	3 (7.7%)	
***Follow-up II***				
** SSQ**	≥ 0.48 (*n* = 102)	56 (100.0%)	46 (100.0%)	-
** HAM-A**	0–7 (*n* = 83)	45 (80.4%)	38 (82.6%)	0.342
	8–14 (*n* = 16)	10 (17.9%)	6 (13.0%)	
	15–23 (*n* = 1)	1 (1.8%)	0 (0.0%)	
	≥ 24 (*n* = 2)	0 (0.0%)	2 (4.3%)	
** BPRS**	0–31 (*n* = 77)	43 (95.6%)	34 (89.5%)	0.548
	32–41 (*n* = 3)	1 (2.2%)	2 (5.3%)	
	42–53 (*n* = 3)	1 (2.2%)	2 (5.3%)	
***Follow-up III***				
** SSQ**	≥ 0.48 (*n* = 102)	55 (100.0%)	45 (100.0%)	-
** HAM-A**	0–7 (*n* = 85)	45 (81.8%)	40 (88.9%)	0.277
	8–14 (*n* = 12)	9 (16.4%)	3 (6.7%)	
	15–23 (*n* = 2)	1 (1.8%)	1 (2.2%)	
	≥ 24 (*n* = 1)	0 (0.0%)	1 (2.2%)	
** BPRS**	0–31 (*n* = 78)	42 (95.5%)	36 (94.7%)	0.169
	32–41 (*n* = 2)	2 (4.5%)	0 (0.0%)	
	42–53 (*n* = 2)	0 (0.0%)	2 (5.3%)	

*BRV* Brivaracetam, *BPRS* Brief psychiatric rating scale, *HAM-A* Hamilton anxiety rating scale, *LEV* Levetiracetam, *SSQ*Seizure severity questionnaire

Both BAEs and non-behavioural adverse events (NBAEs) were assessed and compared among the patients in both treatment groups and different age groups at baseline and at three subsequent follow-ups. Among the 115 patients at baseline, the most commonly observed BAEs were anxiety (65.2%), aggression (55.7%), tension (59.1%), and depression (49.6%). At baseline, adults taking LEV showed a higher percentage of BAEs compared to those taking BRV. We observed a significantly higher percentage of drowsiness and intellectual problems in the BRV group. In pediatric patients, higher percentages of aggression (*P* = 0.089*)* and drowsiness (*P* = 0.028) were observed in patients taking BRV than in those taking LEV. Among the different age groups, statistically significant associations were observed for depression (*P* = 0.052) and tension (*P* = 0.044) with LEV and aggression (*P* = 0.054) with BRV pediatric patients. A higher percentage of BAEs was observed in males compared to females, with significant differences in headache (*P* = 0.013) and depression (*P* = 0.034) for LEV between sexes (Table 4).

**Table 4:** Comparison of BAEs and NBAEs in adults and pediatric patients receiving LEV and BRV at baseline

Side effects	Adult patients (*n* = 95)			Pediatric patients (*n* = 20)		
	**LEV (*n* = 55)**	**BRV (*n* = 40)**	***P*-value**	**LEV (*n* = 11)**	**BRV (*n* = 9)**	***P*-** **value**
Aggression	29 (52.7%)	18 (45.0%)	0.457	8 (72.7%)	9 (100.0%)	0.089
Anxiety	37 (67.3%)	29 (72.5%)	0.585	6 (54.5%)	3 (33.3%)	0.343
Fear	17 (30.9%)	13 (32.5%)	0.869	2 (18.2%)	3 (33.3%)	0.436
Tension	34 (61.8%)	26 (65.0%)	0.751	4 (36.4%)	4 (44.4%)	0.714
Hallucination	4 (7.3%)	5 (12.5%)	0.390	0 (0.0%)	0 (0.0%)	-
Depression	29 (52.7%)	23 (57.5%)	0.644	1 (9.1%)	4 (44.4%)	0.069
Suicidal ideation	0 (0.0%)	0 (0.0%)	-	0 (0.0%)	0 (0.0%)	-
Drowsiness	12 (21.8%)	14 (35.0%)	0.155	2 (18.2%)	6 (66.7%)	**0.028**
Intellectual problems	14 (25.5%)	15 (37.5%)	0.208	7 (63.6%)	5 (55.6%)	0.714
Headache	17 (30.9%)	10 (25.0%)	0.528	0 (0.0%)	1 (11.1%)	0.257
Insomnia	16 (29.1%)	11 (27.5%)	0.865	1 (9.1%)	0 (0.0%)	0.353

*BRV* Brivaracetam, *LEV* Levetiracetam

The main TEAEs observed in the follow up I (*n* = 103) adult LEV group (*n* = 45) were tension (60.0%), fear (60.0%), and anxiety (57.8%). For the adult BRV group (*n* = 39), the most frequent TEAEs were aggression (33.3%), fear (53.8%), and tension (48.7%). In the pediatric LEV group (*n* = 11), the primary TEAEs were aggression (36.4%), anxiety (36.4%), and fear (27.3%). For the pediatric BRV group (*n *= 8), drowsiness (50.0%), aggression (62.5%), and fear (37.5%) were the most prominent. A statistically significant difference was observed for drowsiness (*P *= 0.046) in pediatric patients (Table 5).

**Table 5:** Comparison of BAEs and NBAEs in adults and pediatric patients receiving LEV and BRV at Follow-up I

**Side effects**	**Adult patients** **(*n* = 84)**		***P*- value**	**Side effects**	**Pediatric patients** **(*n* = 19)**		***P*- value**
	**LEV** **(*n* = 45)**	**BRV** **(*n* = 39)**			**LEV** **(*n* = 11)**	**BRV** **(*n* = 8)**	
Aggression (*n* = 32)	19 (42.2%)	13 (33.3%)	0.403	Aggression (*n* = 9)	4 (36.4%)	5 (62.5%)	0.260
Anxiety (*n* = 44)	26 (57.8%)	18 (46.2%)	0.287	Anxiety (*n* = 6)	4 (36.4%)	2 (25.0%)	0.599
Fear (*n* = 48)	27 (60.0%)	21 (53.8%)	0.570	Fear (*n* = 6)	3 (27.3%)	3 (37.5%)	0.636
Tension (*n* = 46)	27 (60.0%)	19 (48.7%)	0.300	Tension (*n* = 5)	2 (18.2%)	3 (37.5)	0.345
Hallucination (*n* = 4)	2 (4.4%)	2 (5.1%)	0.883	Hallucination (*n* = 19)	0 (0.0%)	0 (0.0%)	-
Depression (*n* = 38)	21 (46.7%)	17 (43.6%)	0.778	Depression (*n* = 5)	3 (27.3%)	2 (25.0%)	0.912
Suicidal intension (*n* = 1)	0 (0.0%)	1 (2.6%)	0.280	Suicidal intension (*n* = 0)	0 (0.0%)	0 (0.0%)	-
Drowsiness (*n* = 12)	4 (8.9%)	8 (20.5%)	0.129	Drowsiness (*n* = 5)	1 (9.1%)	4 (50.0%)	**0.046**
Intellectual (*n* = 13)	6 (13.3%)	7 (17.9%)	0.560	Intellectual (*n* = 2)	2 (18.2%)	0 (0.0%)	0.202
Headache (*n* = 2)	1 (2.2%)	1 (2.6%)	0.918	Headache (*n* = 1)	0 (0.0%)	1 (12.5%)	0.228
Insomnia (*n* = 10)	8 (17.8%)	2 (5.1%)	0.074	Insomnia (*n* = 1)	1 (9.1%)	0 (0.0%)	0.381

*BRV* Brivaracetam, *LEV* Levetiracetam

The main TEAEs observed in the follow up II (*n* = 102) for adult LEV group (*n* = 45) were fear (57.8%), anxiety (53.3%), and tension (51.1%). For the adult BRV group (*n* = 38), the most frequent TEAEs were fear (50.0%), tension (42.1%), and depression (39.5%). In the pediatric LEV group (*n* = 11), the primary TEAEs were aggression (54.5%), anxiety (36.4%), and fear (36.4%). For the pediatric BRV group (*n* = 8), drowsiness (50.0%), fear (50.0%), and aggression (62.5%) were the most prominent. A statistically significant difference was observed for anxiety (*P* = 0.006) in adult patients and for drowsiness (*P* = 0.060) in pediatric patients (Table 6).

**Table 6:** Comparison of BAEs and NBAEs in adults and pediatric patients receiving LEV and BRV at Follow-up II

**Side effects**	**Adult patients** **(*n* = 83)**		***P*- value**	**Side effects**	**Pediatric patients** **(*n* = 19)**		***P*- value**
	**LEV** **(*n* = 45)**	**BRV** **(*n* = 38)**			**LEV** **(*n* = 11)**	**BRV** **(*n* = 8)**	
Aggression (*n* = 28)	17 (37.8%)	11 (28.9%)	0.397	Aggression (*n* = 11)	6 (54.5%)	5 (62.5%)	0.729
Anxiety (*n* = 33)	24 (53.3%)	9 (23.7%)	**0.006**	Anxiety (*n* = 6)	4 (36.4%)	2 (25.0%)	0.599
Fear (*n* = 45)	26 (57.8%)	19 (50.0%)	0.479	Fear (*n* = 8)	4 (36.4%)	4 (50.0%)	0.552
Tension (*n* = 39)	23 (51.1%)	16 (42.1%)	0.413	Tension (*n* = 13)	2 (18.2%)	4 (50.0%)	0.141
Hallucination (*n* = 5)	2 (4.4%)	3 (7.9%)	0.510	Hallucination (*n* = 0)	0 (0.0%)	0 (0.0%)	-
Depression (*n* = 34)	19 (42.2%)	15 (39.5%)	0.800	Depression (*n* = 4)	3 (27.3%)	1 (12.5%)	0.435
Suicidal intension (*n* = 1)	0 (0.0%)	1 (2.6%)	0.274	Suicidal intension (*n* = 0)	0 (0.0%)	0 (0.0%)	-
Drowsiness (*n* = 12)	5 (11.1%)	7 (18.4%)	0.345	Drowsiness (*n* = 4)	0 (0.0%)	4 (50.0%)	**0.008**
Intellectual (*n* = 10)	5 (11.1%)	5 (13.2%)	0.775	Intellectual (*n* = 14)	8 (72.7%)	6 (75.0%)	0.912
Headache (*n* = 2)	1 (2.2%)	1 (2.6%)	0.904	Headache (*n* = 1)	0 (0.0%)	1 (12.5%)	0.228
Insomnia (*n* = 8)	5 (11.1%)	3 (7.9%)	0.621	Insomnia (*n* = 2)	1 (9.1%)	1 (12.5%)	0.811

*Abbreviation: BRV* Brivaracetam, *LEV* Levetiracetam

The main TEAEs observed in the follow up III (*n *= 100) for adult LEV group (*n *= 44) were tension (52.3%), anxiety (43.2%), and aggression (40.9%). In the adult BRV group (*n *= 38), the most frequent TEAEs were tension (36.8%), depression (34.2%), and drowsiness (21.1%). For the pediatric LEV group (*n *= 11), the primary TEAEs were aggression (45.5%) and fear (27.3%). In the pediatric BRV group (*n *= 7), drowsiness (57.1%), aggression (57.1%), and anxiety (28.6%) were the most prominent. A statistically significant difference was observed for drowsiness (*P *= 0.004) in pediatric patients (Table 7).

**Table 7:** Comparison of BAEs and NBAEs in adults and pediatric patients receiving LEV and BRV at Follow-up III

Side effects	Adult patients (*n* = 82)		*P*- value	Side effects	Pediatric patients (*n* = 18)		*P*- value
	**LEV** **(*n* = 44)**	**BRV** **(*n* = 38)**			**LEV** **(*n* = 11)**	**BRV** **(*n* = 7)**	
Aggression (*n* = 26)	18 (40.9%)	8 (21.1%)	0.054	Aggression (*n* = 9)	5 (45.5%)	4 (57.1%)	0.629
Anxiety (*n* = 28)	19 (43.2%)	9 (23.7%)	0.063	Anxiety (*n* = 4)	2 (18.2%)	2 (28.6%)	0.605
Fear (*n* = 18)	11 (25.0%)	7 (18.4%)	0.473	Fear (*n* = 5)	3 (27.3%)	2 (28.6%)	0.952
Tension (*n* = 37)	23 (52.3%)	14 (36.8%)	0.161	Tension (*n* = 6)	2 (18.2%)	4 (57.1%)	0.087
Hallucination (*n* = 4)	1 (2.3%)	3 (7.9%)	0.239	Hallucination	0 (0.0%)	0 (0.0%)	-
Depression (*n* = 28)	15 (34.1%)	13 (34.2%)	0.991	Depression (*n* = 4)	3 (27.3%)	1 (14.3%)	0.518
Suicidal intension (*n* = 1)	0 (0.0%)	1 (2.6%)	0.279	Suicidal intension	0 (0.0%)	0 (0.0%)	-
Drowsiness (*n* = 13)	5 (11.4%)	8 (21.1%)	0.231	Drowsiness (*n* = 4)	0 (0.0%)	4 (57.1%)	**0.004**
Intellectual (*n* = 10)	4 (9.1%)	6 (15.8%)	0.355	Intellectual (*n* = 4)	2 (18.2%)	2 (28.6%)	0.605
Headache (*n* = 3)	2 (4.5%)	1 (2.6%)	0.645	Headache (*n* = 1)	0 (0.0%)	1 (14.3%)	0.197
Insomnia (*n* = 7)	5 (11.4%)	2 (5.3%)	0.324	Insomnia (*n* = 2)	1 (9.1%)	1 (14.3%)	0.197

*Abbreviation*: *BRV* Brivaracetam, *LEV* Levetiracetam

Among the 115 patients included in the study, 16 (13.9%) were assessed for treatment emergent hematological abnormalities, including 14 patients (87.5%) receiving LEV (13 adults and 1 pediatric patient), and 2 patients (12.5%) receiving BRV (1 adult and 1 pediatric patient respectively). The most common hematological abnormalities observed in patients taking LEV were eosinopenia, increased polymorphs, monocytopenia, lymphopenia, and decreased red blood cell (RBC) count. All of these findings were observed in adult patients. However, the most common hematological abnormalities observed in patients receiving BRV was increased polymorphs (100% in both adult and pediatric patients), decreased packed cell volume (PCV) in adult patients and lymphopenia in pediatric patients (Table 8). The limited number of cohorts available for assessing hematological abnormalities is primarily due to the loss of follow-up and patient non-cooperation.

**Table 8:** Safety analysis and comparison of the hematological side effects observed in both LEV and BRV in adult and pediatric patients

Parameters	LEV (Adult, *n* = 13)	BRV (Adult, *n* = 1)	LEV (Pediatric, *n* = 1)	BRV (Pediatric, *n* = 1)
Decreased RBC	5 (38%)	1	0	0
Increased total count	3 (23%)	1	0	0
Decreased total count	1 (7.6%)	0	0	0
Increased polymorphs	8 (62%)	1	0	1
Eosinopenia	9 (69%)	0	0	0
Monocytopenia	5 (38%)	0	1	0
Decreased hemoglobin	1 (7.6%)	0	0	0
Lymphopenia	5 (38%)	0	0	1
Lymphocytosis	3 (23%)	0	0	0
PCV decreased	3 (23%)	1	0	0
MCH decreased	2 (15.3%)	0	0	0

*BRV* Brivaracetam, *LEV* Levetiracetam, *RBC* Red blood cells, *PCV* Packed cell volume, *MCH* Mean corpuscular hemoglobin

During the study period, in follow-up I (*n* = 103), 8 patients had a switch from their existing antiepileptic therapy. Among them, 5 patients switched from LEV to BRV, and 3 patients switched from BRV to LEV. The main reason for the switch from LEV to BRV was the BAEs. Drowsiness, the most common side effect in patients taking BRV, was responsible for the switch from BRV to LEV (Table S1). No patients switched treatments in follow-up II (*n* = 102). In follow-up III (*n* = 100), one patient who had initially switched from LEV to BRV in follow-up I switched back to LEV due to seizure recurrence on BRV (Table S2). The patients who switched from LEV to BRV had significant improvements in side effects and seizure frequency, whereas one of the patients had poor tolerability to BRV. The mean SSQ score was 0.49 ± 0.015. The mean scores for HAM-A and BPRS were 10.21 ± 0.76 and 31.58 ± 1.43, respectively. The mean score changes were also compared between treatment arms. The mean scores at follow-up I for the SSQ, HAM-A, and BPRS were 0.95 ± 0.014, 6.14 ± 0.636, and 25.26 ± 0.91 respectively. The mean scores at follow-up II for the SSQ, HAM-A, and BPRS were 0.99 ± 0.006, 4.42 ± 0.50, and 22.92 ± 0.77, respectively. However, at follow-up III, the mean scores for the SSQ, HAM-A, and BPRS were 0.995 ± 0.004, 3.98 ± 0.46, and 21.94 ± 0.70, respectively. For the PESQ, the means were 22.36 ± 4.41, 11.0 ± 3.73, 9.63 ± 2.41 and 9.78 ± 2.94 at baseline, and at follow-ups I, II, and III, respectively (Table S3, Table S4, Table S5 and Table S6). The TEAEs in pediatric patients receiving LEV or BRV showed no severe AEs in either group. The highest frequency of TEAEs, with moderate severity, was reported in the LEV group. Overall, all assessed scores ( seizure severity and safety measures) showed a declining trend.

At different LEV and BRV doses, the frequency of TEAEs varied (Table S7, S8). In this study, 100 mg BRV resulted in a relatively high percentage of BAEs, while the highest percentage of BAEs was reported in LEV 1000 mg. The mean daily doses were 1000 mg for LEV and 100mg for BRV. All the patients who were assessed in follow-up III (*n* = 100) were compared to their baseline data (with the same patients compared at both time points) to establish a clinical correlation of the efficacy and TEAEs observed for the respective treatment groups. The results of this comparison yielded clinically and statistically significant findings (Table 9, Table 10). Patients who switched from LEV to BRV (*n* = 3) or from BRV to LEV (*n* = 2) were compared to their baseline values separately (Table 11, Table 12).

**Table 9:** Comparison of AEs between baseline and follow-up III among the LEV group (*n* = 53)

Baseline AE (*n* = 53)	Follow-up III AE (*n* = 53)		*P*-value
	**AE that persists in follow-up III**	**No AE in** **follow-up III**	
Aggression (*n* = 31) No aggression(*n* = 22)	18 (58.0%) 3 (13.6%)	13 (42.0%) 19 (86.4%)	**0.001**
Headache (*n* = 13) No headache (*n* = 40)	1 (7.7%) 0 (0.0%)	12 (92.3%) 40 (100.0%)	0.077
Drowsiness (*n* = 11) No drowsiness (*n* = 42)	1 (9.0%) 4 (9.5%)	10 (91.0%) 38 (90.5%)	0.965
Anxious (*n* = 36) No anxious (*n* = 17)	20 (55.6%) 0 (0.0%)	16 (44.4%) 17(100.0%)	**-**
Tension (*n* = 30) No tension (*n* = 23)	19 (63.3%) 4 (17.4%)	11 (36.7%) 19 (82.6%)	**0.001**
Fear (*n* = 15) No fear (*n* = 38)	8 (53.3%) 5 (13.1%)	7 (46.7%) 33 (86.9%)	**0.002**
Insomnia (*n* = 11) No insomnia (*n* = 42)	2 (18.1%) 2 (4.8%)	9 (81.9%) 40 (95.2%)	0.134
Depression (*n* = 25) No depression (*n* = 28)	11 (44%) 5 (17.9%)	14 (56%) 23 (82.1%)	**0.038**
Hallucination (*n* = 2) No hallucination(*n* = 51)	0 (0.0%) 1 (1.9%)	2 (100.0%) 50 (98.1%)	0.842
Suicidal ideation No suicidal ideation	0 (0.0%) 0 (0.0%)	0 (0.0%) 0 (0.0%)	-
Intellectual (*n* = 17) No intellectual (*n* = 36)	15 (88.2%) 33 (91.7%)	2 (11.8%) 3 (8.3%)	0.690

*AE* Adverse events

**Table 10:** Comparison of AEs between baseline and follow-up III among the BRV group (*n* = 42)

Baseline AE (*n* = 42)	Follow-up III AE (*n* = 42)		*P*-value
	**AE that persists in follow-up III**	**No AE in** **follow-up III**	
Aggression (*n* = 23) No aggression(*n* = 19)	10 (43.5%) 1 (5.3%)	13 (56.5%) 18 (94.7%)	**0.005**
Headache (*n* = 8) No headache(*n* = 34)	1 (12.5%) 1 (3.0%)	7 (87.5%) 33 (97.0%)	0.253
Drowsiness (*n* = 17) No drowsiness(*n* = 25)	7 (41.1%) 4 (16.0%)	10 (58.9%) 21 (84.0%)	**0.069**
Anxious (*n* = 28) No anxious(*n* = 14)	10 (35.7%) 0 (0.0%)	18 (64.3%) 14 (100.0%)	**0.010**
Tension (*n* = 25) No tension (*n* = 17)	16 (64.0%) 1 (5.9%)	9 (36.0%) 16 (94.1%)	**0.000**
Fear (*n* = 15) No fear(*n* = 27)	7 (46.7%) 1 (3.7%)	8 (53.3%) 26 (96.3%)	**0.001**
Insomnia (*n* = 7) No insomnia(*n* = 35)	2 (28.5%) 1 (2.9%)	5 (71.5%) 34 (97.1%)	**0.016**
Depression (*n* = 23) No depression (*n* = 19)	12 (52.2%) 1 (5.3%)	11 (47.8%) 18 (94.7%)	**0.001**
Hallucination (*n* = 4) No hallucination(*n* = 38)	2 (50.0%) 0 (0.0%)	2 (50.0%) 38 (100.0%)	**-**
Suicidal ideation No suicidal ideation	0 (0.0%) 0 (0.0%)	0 (0.0%) 0 (0.0%)	**-**
Intellectual (*n* = 16) No intellectual(*n* = 26)	10 (62.5%) 24 (92.3%)	6 (37.5%) 2 (7.7%)	**0.017**

*Abbreviation: AE* Adverse events

**Table 11:** Comparison of AEs between baseline and follow-up III among the BRV and LEV groups (*n* = 2)

Baseline AE (*n* = 2)	Follow-up III AE (*n* = 2)		*P*-value
	**AE that persists in follow- up III**	**No AE in** **follow-up III**	
Aggression (*n* = 1) No aggression (*n* = 1)	1 (100.0%) 1 (100.0%)	0 (0.0%) 0 (0.0%)	-
Headache (*n* = 2) No headache (*n* = 0)	1 (50.0%) 0 (0.0%)	1 (50.0%) 0 (0.0%)	-
Drowsiness (*n* = 1) No drowsiness(*n* = 1)	0 (0.0%) 0 (0.0%)	1 (100.0%) 1 (100.0%)	-
Anxious (*n* = 2) No anxious(*n* = 0)	1 (50.0%) 0 (0.0%)	1 (50.0%) 0 (0.0%)	-
Tension (*n* = 2) No tension(*n* = 0)	2 (100.0%) 0 (0.0%)	0 (0.0%) 0 (0.0%)	-
Fear (*n* = 1) No fear(*n* = 1)	0 (0.0%) 1 (100.0%)	1 (100.0%) 0 (0.0%)	0.157
Insomnia (*n* = 2) No insomnia(*n* = 0)	2(100.0%) 0 (0.0%)	0 (0.0%) 0 (0.0%)	-
Depression (*n* = 2) No depression(*n* = 0)	2 (100.0%) 0 (0.0%)	0 (0.0%) 0 (0.0%)	-
Hallucination (*n* = 1) No hallucination(*n* = 1)	0 (0.0%) 0 (0.0%)	1 (100.0%) 1 (100.0%)	-
Suicidal ideation No suicidal ideation	0 (0.0%) 0 (0.0%)	0 (0.0%) 0 (0.0%)	-
Intellectual (*n* = 2) No intellectual(*n* = 0)	1 (50.0%) 0 (0.0%)	1 (50.0%) 0 (0.0%)	-

*AE* Adverse events

**Table 12:** Comparison of AEs between baseline and follow-up III among the LEV and BRV groups (*n* = 3)

AE (*n* = 3)	Follow-up III AE (*n* = 3)		*P*-value
	**AE that persists** **in follow-up III**	**No AE in** **follow-up III**	
Aggression (*n* = 2) No aggression (*n* = 1)	0 (0.0%) 1 (100.0%)	2 (100.0%) 0 (0.0%)	0.083
Headache (*n* = 1) No headache(*n* = 2)	0 (0.0%) 0 (0.0%)	1(100.0%) 2 (100.0%)	-
Drowsiness (*n* = 1) No drowsiness(*n* = 2)	0 (0.0%) 1 (50.0%)	1 (100.0%) 1 (50.0%)	0.386
Anxious (*n* = 2) No anxious(*n* = 1)	1 (50.0%) 0 (0.0%)	1 (50.0%) 1 (100.0%)	0.386
Tension (*n* = 2) No tension(*n* = 1)	1 (50.0%) 0 (0.0%)	1 (50.0%) 1 (100.0%)	0.386
Fear (*n* = 1) No fear(*n* = 2)	1 (100.0%) 0 (0.0%)	0 (0.0%) 2 (100.0%)	0.083
Insomnia No insomnia	0 (0.0%) 0 (0.0%)	0 (0.0%) 0 (0.0%)	-
Depression (*n* = 1) No depression(*n* = 2)	0 (0.0%) 1 (50.0%)	1 (100.0%) 1 (50.0%)	0.386
Hallucination (*n* = 0) No hallucination(*n* = 3)	0 (0.0%) 1 (33.3%)	0 (0.0%) 2 (66.7%)	-
Suicidal ideation No suicidal ideation	0 (0.0%) 0 (0.0%)	0 (0.0%) 0 (0.0%)	-
Intellectual No intellectual	0 (0.0%) 0 (0.0%)	0 (0.0%) 0 (0.0%)	-

*AE* Adverse events

## Discussion

This study assessed the effectiveness and safety profiles of LEV (*n* = 63) and BRV (*n* = 52) in a cohort of 115 patients with different types of seizures. The prospective design and objective evaluation of behavioural and non-behavioural side effects, hematological abnormalities, as well as the assessment switching between LEV and BRV, are the differential factors of this research. While it is undeniable that there are considerable differences both within and between the LEV and BRV groups, the results suggest that, in terms of TEAE, BRV is superior to LEV and appears to be a safer option. The safety and effectiveness of BRV and LEV, as well as switching from LEV to BRV, have been independently evaluated in previous publications. In contrast, this study uniquely examines both cohorts concurrently, focusing on the Indian population and BRV to LEV switching as well. Based on the results of this study, LEV has shown better efficacy in reducing overall seizure frequency than BRV. One of the striking results was the treatment of emergent BAEs on BRV, which influenced patients' medication adherence. We observed that patients taking BRV also experienced side effects comparable to those taking LEV. Nonetheless, patients reported fewer and less severe TEAEs with both BRV and the switch from LEV to BRV compared to LEV alone, supporting BRV's clinical superiority in terms of TEAEs reduction. There was also a significant reduction (*P* = 0.002) in their overall scores during the follow-ups. Drowsiness was the most common NBAEs reported in the BRV group (25%, 26.1%, and 23% in follow-up I, II, and III, respectively). In contrast, LEV-treated patients reported lower rates (8%, 9.3%, and 8.9% in follow- up I, II, and III, respectively). According to other publications, the prevalence of drowsiness during BRV use has been highlighted [14, 15]. Randomized controlled trials (RCTs) [16–18] along with retrospective studies [19–22] show that drowsiness observed in patients taking BRV ranges from 4% to 17%. A meta-analysis of RCTs [23] identified drowsiness to be one of the three most frequent TEAEs with BRV, alongside being fatigue and back pain. The most common BAEs observed with LEV was suicidal ideation. A literature review [24] and a cohort study [25] revealed that LEV-associated suicidal ideation is an alarming matter of debate. In contrast, the present study reported that no patients experienced suicidal ideation while taking LEV; rather, one patient (1.92%) on BRV (*n* = 52) experienced suicidal ideation with no previous history of suicidal thoughts. However, the severity of this symptom decreased during the final follow-up of the study. Psychiatric side effects can prompt treatment discontinuation and restrict the LEV dose range, particularly in patients with psychiatric comorbidities or mental disabilities [26]. Another prominent aspect of this study is that it has distinctly compared the safety of LEV and BRV in both adult and pediatric populations. The results indicate fewer BAEs associated with LEV in the pediatric population (*n* = 19) compared to BRV. Additionally, pediatric patients on BRV experienced a significant greater reduction in BAEs. The most common TEAEs associated with BRV among pediatric patients were aggression, anxiety, and drowsiness. However, pediatric patients taking LEV had higher incidences of aggression, depression, and intellectual impairment. In two pediatric patients, BRV was associated with poor seizure control, which caused one to switch from BRV to LEV. However, overall, LEV and BRV achieved similar seizure control among the other patients. BRV demonstrated superior affinity and selectivity for the SV2A receptor in comparison with LEV. These findings suggest that BRV may be more effective in reducing seizures. However, there were no significant differences in clinical efficacy between the two groups in this study. Remarkably, even among patients who positively responded to LEV, not all of them exhibited a similar response after switching to BRV. In the follow-up (*n* = 100), 2% showed poor seizure control on BRV. This unexpected finding suggests that higher affinity for the SV2A receptor may not directly improve the efficacy of BRV. To provide a definitive statement on this matter, further research with a larger sample size comparing LEV and BRV is needed. A retrospective review by Tekgül et al. [27], Hirsch et al. [28], and a meta-analysis by Verrotti et al. [29] revealed that there was no correlation between the LEV dose and side effects. Compared with other doses, LEV had greater side-effect incidence at 1000 mg/day, whereas BRV exhibited higher TEAE rates at 100 mg/day. The rationale for this observation is unknown, but the fact that the BAEs (observed for a few weeks to months) suggests they may not be dose-dependent and may involve some underlying neurological pathology. Although LEV and BRV share a similar mechanism of action involving the SV2A protein, recent research conducted by Wood and Gillard [30] suggested that they may target different binding sites on the SV2A protein. Hirsch et al. [28] have postulated that this difference in binding sites could account for the improved seizure control observed in BRV therapy. This may explain why approximately 80% of patients who switched from LEV to BRV achieved better seizure control and fewer side effects. This aligns with the findings of a multicenter retrospective study conducted by Villanueva et al. [31], where 55.6% of LEV non-responders experienced seizure reduction after switching to BRV. However, findings from the present study and previous clinical investigations indicate that switching to BRV can be advantageous when BAEs are encountered while LEV therapy is used. In this study, patients experienced decreased TEAEs after switching from LEV to BRV, which aligns with the results of clinical studies conducted by Yates et al. [15] and Steinig et al. [22]. Hematological abnormalities associated with LEV and BRV are rare. For LEV, a report by Alzahrani et al. [32] suggested hematological abnormalities are typically limited to mild cases of thrombocytopenia, leukopenia, or anemia. In comparison, limited observational studies have explored hematological abnormalities linked to BRV. The most common hematological abnormalities observed in patients taking LEV (14/16, 87.5%) included eosinopenia, increased polymorphs, monocytopenia, lymphopenia, and decreased RBC counts. In contrast to studies by Bachmann et al. [33] and Sahaya et al. [34], which reported an association between LEV and hematological abnormalities such as thrombocytopenia and pancytopenia; however, no such cases were observed here . For BRV, the most common hematological abnormalities (12.7%) were increased polymorphs, decreased packed cell volume (PCV) and lymphopenia. In a study by Brandt et al. [35], the common hematological abnormalities associated with BRV were neutropenia, followed by leukopenia and lymphopenia, which closely corresponds with our results. Interestingly, among the patients undergoing hematological abnormalities assessment, the most prominent AE with LEV was eosinopenia, which is extremely rare, while BRV was associated with increased polymorphs and lymphopenia, which are also rarely reported and studied. The exact mechanism is still unknown. All patients who were assessed for hematological abnormalities received LEV or BRV as monotherapy without concurrent ASMs. This suggests a potential causal link to LEV or BRV. Additional studies with larger cohorts are necessary to establish the mechanism and validate these results. After evaluating questionnaire scores separately at baseline and three follow-ups, we observed a decrease in mean score for both groups. Direct comparison of LEV and BRV using individual assessment tools revealed comparable safety profiles, with both groups showing statistically significant (*P* < 0.05) decrease in seizure severity. The results almost align with a previous study [36], which stated that there were no significant differences between LEV and BRV regarding efficacy, or safety (including BAEs and NBAEs), except for drowsiness, which was more frequent with high-dose BRV. In follow-up III, comparison with baseline data revealed both clinically and statistically significant values, which confirms that LEV and BRV may be associated with BAEs and NBAEs in patients with epilepsy, thereby aiding clinicians in devising a comprehensive therapeutic strategy for prescribing these medications.

### Limitations

Our study still has several limitations. Potential bias may exist from self-reported adverse effects. Although we collected data regarding daily doses of LEV and BRV, medication adherence could not be assessed. A responder-rate comparison between LEV and BRV was not feasible, as only descriptive counts were obtained—a limitation that should be considered when interpreting these findings. Due to the short study duration, loss to follow-up and lack of cooperation from the patients, very few samples were collected to analyze hematological abnormalities. Additionally, the sample size for patients switching between LEV and BRV was limited, due to the relatively lower occurrence of such transitions in clinical practice. However, despite the smaller cohort, the study provides valuable insights into the tolerability and effectiveness of these switches, contributing to existing literature on real-world treatment modifications.

## Conclusions

In conclusion, this study indicates that BRV in epileptic patient results in fewer and less sever BAEs compared to LEV. The study also suggested that LEV may have slightly higher efficacy and a lower incidence of drowsiness than BRV, where drowsiness was the most frequently reported AE. On the other hand, BRV appears to be safer and more tolerable for patients with LEV-induced BAEs. In pediatric population, both LEV and BRV showed similar efficacy, with relatively fewer BAEs associated with LEV. The majority of the patients who switched from LEV to BRV due to BAEs or poor seizure control on LEV had significant improvement after initiation of BRV (with or without titration). However, this study also included patients who experienced refractory seizures with BRV when they switched from LEV to BRV. Hematological abnormalities observed in both LEV and BRV groups are very rare, but their potential association should not be overlooked. Overall, BRV may be a safer option than LEV for managing seizures. Therefore, these findings underscore the importance of conducting a comprehensive psychiatric evaluation of patients prior to the initiation of treatment with LEV or BRV to optimize safety. Additional prospective studies are warranted to further validate these results.

## Supplementary Information

Additional file 1: Additional information includes Table S1: Adverse events (AEs) observed when patients switched from LEV to BRV or BRV to LEV in follow- up I. Table S2: AEs observed in patients when switched from BRV to LEV in follow-up III. Table S3: Baseline score distribution among LEV and BRV. Table S4: Follow-up I score distribution among LEV and BRV. Table S5: Follow-up II score distribution among LEV and BRV and Table S6: Follow-up III score distribution among LEV and BRV. Table S7: TEAEs in different dose ranges among BRV patients. Table S8: TEAEs in different dose ranges among LEV patients.

## Data Availability

The data and supporting materials used during the current study have been submitted for storage and data are available from the corresponding author upon reasonable request.

## Abbreviations

AE: Adverse event
ASMs: Anti-seizure medications
BAEs: Behavioural adverse events
BRV: Brivaracetam
BPRS: Brief Psychiatric Rating Scale
HAM-A: Hamilton Anxiety Rating Scale
LEV: Levetiracetam
NBAEs: Non-behavioural adverse events
PESQ: Pediatrics Epilepsy Side Effects Questionnaire
SSQ: Seizure Severity Questionnaire
SV2A: Synaptic vesicle protein 2A
TEAE: Treatment-emergent adverse events
