# Supplementary material for: Effectiveness and safety of brivaracetam in comparison with levetiracetam in seizures
Source: Acta Epileptol. 2025 Oct 2;7:47. doi: 10.1186/s42494-025-00229-z (PMC12490144; doi:10.1186/s42494-025-00229-z)
Supplement: Supplementary file 1 — Additional file 1: Additional information includes Table S1: Adverse events (AEs) observed when patients switched from LEV to BRV or BRV to LEV in follow- up I. Table S2: AEs observed in patients when switched from BRV to LEV in follow-up III. Table S3: Baseline score distribution among LEV and BRV. Table S4: Follow-up I score distribution among LEV and BRV. Table S5: Follow-up II score distribution among LEV and BRV and Table S6: Follow-up III score distribution among LEV and BRV. Table S7: TEAEs in different dose ranges among BRV patients. Table S8: TEAEs in different dose ranges among LEV patients. [file 42494_2025_229_MOESM1_ESM.docx]

**Supplementary Tables**

**Table S1** Adverse events **(**AEs) observed when patients switched from LEV to BRV or BRV to LEV in follow- up I

| **Side effects** | **BRV to LEV (*n*=3)** | **LEV to BRV (*n*=5)** | ***P*- value** |
| --- | --- | --- | --- |
| Aggression | 2 (66.7%) | 2 (40%) | 0.465 |
| Anxiety | 1 (33.3%) | 2 (40%) | 0.850 |
| Tension | 3 (100%) | 1 (20%) | **0.028** |
| Fear | 0 | 1 (20%) | 0.408 |
| Depression | 2 (66.7%) | 2 (40%) | 0.465 |
| Drowsiness | 1 (33.3%) | 2 (40%) | 0.850 |
| Intellectual | 1(33.3%) | 0 | 0.168 |
| Insomnia | 2 (66.7%) | 0 | 0.035 |

BRV: Brivaracetam, LEV: Levetiracetam

**Table S2** AEs observed in patients when switched from BRV to LEV in follow-up III

| **Side effects** | **BRV to LEV (*n*=1)** |
| --- | --- |
| Aggression | 1 (100%) |
| Tension | 0 |
| Fear | 0 |
| Hallucination | 0 |
| Depression | 0 |
| Suicidal intention | 0 |
| Drowsiness | 0 |
| Intellectual | 0 |
| Headache | 0 |
| Insomnia | 0 |

BRV: Brivaracetam, LEV: Levetiracetam

**Table S3** Baseline score distribution among LEV and BRV

| **SCORE** |  | **LEV (*n*=66)** | **BRV (*n*=49)** | ***P*-value** |
| --- | --- | --- | --- | --- |
| SSQ | 0-0.47  (*n*=54) | 39  (59.1%) | 15  (30.6%) | **0.002** |
|  | 0.48 and  greater (*n*=61) | 27  (40.9%) | 34  (69.4%) |  |
| HAM | 0-7 | 26 | 18 | 0.673 |
|  | (*n*=44) | (39.4%) | (36.7%) |  |
|  | 8-14 | 26 | 16 |  |
|  | (*n*=42) | (39.4%) | (32.7%) |  |
|  | 15-23 | 11 | 11 |  |
|  | (*n*=22) | (16.7%) | (22.4%) |  |
|  | 24 and | 3 | 4 |  |
|  | greater | (4.5%) | (8.2%) |  |
|  | (*n*=7) |  |  |  |
| BPRS | 0-31 | 33 | 24 | 0.754 |
|  | (*n*=57) | (60.0%) | (60.0%) |  |
|  | 32-41 | 15 | 8 |  |
|  | (*n*=23) | (27.3%) | (20.0%) |  |
|  | 42-53 | 3 | 3 |  |
|  | (*n*=6) | (5.5%) | (7.5%) |  |
|  | 54 and | 4 | 5 |  |
|  | greater | (7.3%) | (12.5%) |  |
|  | (*n*=9) |  |  |  |

BRV: Brivaracetam, LEV: Levetiracetam, SSQ: Seizure Severity Questionnaire, HAM: Hamilton Anxiety Scale, BPRS: Brief Psychiatric Rating Scale

**Table S4** Follow-up I score distribution among LEV and BRV

| **SCORE** |  | **LEV (*n*=56)** | **BRV (*n*=47)** | ***P*-value** |
| --- | --- | --- | --- | --- |
| SSQ | 0-0.47 | 1 | 1 | 0.900 |
|  | (*n*=2) | (1.8%) | (2.1%) |  |
|  | 0.48 and | 55 | 46 |  |
|  | greater | (98.2%) | (97.9%) |  |
|  | (*n*=101) |  |  |  |
| HAM | 0-7 (*n*=68) | 35  (62.5%) | 33  (70.2%) | 0.396 |
|  | 8-14  (*n*=25) | 16  (28.6%) | 9  (19.1%) |  |
|  | 15-23 (*n*=7) | 4  (7.1%) | 3  (6.4%) |  |
|  | 24 and greater  (*n*=3) | 1  (1.8%) | 2  (4.3%) |  |
| BPRS | 0-31 (*n*=71) | 37  (82.2%) | 34  (87.2%) | 0.396 |
|  | 32-41  (*n*=8) | 6  (13.3%) | 2  (5.1%) |  |
|  | 42-53  (*n*=5) | 2  (4.4%) | 3  (7.7%) |  |

BRV: Brivaracetam, LEV: Levetiracetam, SSQ: Seizure Severity Questionnaire, HAM: Hamilton Anxiety Scale, BPRS: Brief Psychiatric Rating Scale

**Table S5** Follow-up II score distribution among LEV and BRV

| **SCORE** |  | **LEV (*n*=56)** | **BRV (*n*=46)** | ***P*-value** |
| --- | --- | --- | --- | --- |
| SSQ | 0.48 and  greater (*n*=102) | 56  (100.0%) | 46  (100.0%) | 0 |
| HAM | 0-7 | 45 | 38 | 0.342 |
|  | (*n*=83) | (80.4%) | (82.6%) |  |
|  | 8-14 | 10 | 6 |  |
|  | (*n*=16) | (17.9%) | (13.0%) |  |
|  | 15-23 | 1 | 0 |  |
|  | (*n*=1) | (1.8%) | (0.0%) |  |
|  | 24 and greater | 0 | 2 |  |
|  | (*n*=2) | (0.0%) | (4.3%) |  |
| BPRS | 0-31 | 43 | 34 | 0.548 |
|  | (*n*=77) | (95.6%) | (89.5%) |  |
|  | 32-41 | 1 | 2 |  |
|  | (*n*=3) | (2.2%) | (5.3%) |  |
|  | 42-53 | 1 | 2 |  |
|  | (*n*=3) | (2.2%) | (5.3%) |  |

BRV: Brivaracetam, LEV: Levetiracetam, SSQ: Seizure Severity Questionnaire, HAM: Hamilton Anxiety Scale, BPRS: Brief Psychiatric Rating Scale

**Table S6** Follow-up III score distribution among LEV and BRV

| **SCORE** |  | **LEV (*n*=55)** | **BRV (*n*=45)** | ***P*-value** |
| --- | --- | --- | --- | --- |
| SSQ | 0.48 and | 55 | 45 | 0 |
|  | greater | (100.0%) | (100.0%) |  |
|  | (*n*=102) |  |  |  |
| HAM | 0-7 (*n*=85) | 45  (81.8%) | 40  (88.9%) | 0.277 |
|  | 8-14 (*n*=12) | 9  (16.4%) | 3  (6.7%) |  |
|  | 15-23 (*n*=2) | 1  (1.8%) | 1  (2.2%) |  |
|  | 24 and greater (*n*=1) | 0  (0.0%) | 1  (2.2%) |  |
| BPRS | 0-31 (*n*=78) | 42  (95.5%) | 36  (94.7%) | 0.169 |
|  | 32-41 (*n*=2) | 2  (4.5%) | 0  (0.0%) |  |
|  | 42-53 (*n*=2) | 0  (0.0%) | 2  (5.3%) |  |

BRV: Brivaracetam, LEV: Levetiracetam, SSQ: Seizure Severity Questionnaire, HAM: Hamilton Anxiety Scale, BPRS: Brief Psychiatric Rating Scale

**Table S7** Treatment emergent adverse events in different dose ranges among BRV patients

|  | BRV (*n*=49) | | | | | | | | | Chi-square (*P*-value) |
| --- | --- | --- | --- | --- | --- | --- | --- | --- | --- | --- |
|  | **100 mg** | **150mg** | **175mg** | **2ml** | **200mg** | **25mg** | **5ml** | **50mg** | **75mg** |  |
| Aggression  (*n*=27) | 15  (55.6%) | 3  (11.1%) | 1  (3.7%) | 1  (3.7%) | 2  (7.4%) | 0  (0.0%) | 1  (3.7%) | 3  11.1%) | 1  (3.7%) | 6.598  (0.707) |
| Headache  (*n*=11) | 7  (63.6%) | 2  (18.2%) | 0  (0.0%) | 0  (0.0%) | 1  (9.1%) | 1  (9.1%) | 0  (0.0%) | 0  (0.0%) | 0  (0.0%) | 8.609  (0.333) |
| Drowsiness  (*n*=20) | 12  (60.0%) | 1  (5.0%) | 1  (5.0%) | 0 (0.0%) | 1  (5.0%) | 1  (5.0%) | 0 (0.0%) | 4  (20%) | 0  (0.0%) | 8.559  (0.329) |
| Anxiety  (*n*=32) | 20  (62.5%) | 3  (9.4%) | 0  (0.0%) | 0 (0.0%) | 3  (9.4%) | 1  (3.1%) | 0 (0.0%) | 3  (9.4%) | 2  (6.2%) | 7.964  (0.407) |
| Tension  (*n*=30) | 17  (57.0%) | 3  (10.0%) | 0  (0.0%) | 1 (4.0%) | 3  (10.0%) | 1  (4.0%) | 0  (0.0%) | 3  (4.0%) | 2  (7.0%) | 7.437  (0.505) |
| Fear  (*n*=16) | 8  (50.0%) | 1  (6.3%) | 0  (0.0%) | 1 (6.3% | 2  (12.5%) | 1  (6.3%) | 0 (0.0%) | 3  (18.6%) | 0  (0.0%) | 8.615  (0.318) |
| Insomnia  (*n*=11) | 7  (63.7%) | 0  (0.0%) | 0  (0.0%) | 0 (0.0%) | 0  (0.0%) | 1  (9.0%) | 0 (0.0%) | 2  (18.3%) | 1  (9.0%) | 7.587  (0.464) |
| Intellectual  (*n*=20) | 13  (65.0%) | 2  (10.0%) | 0  (0.0%) | 1 (5.0%) | 0  (0.0%) | 1  (5.0%) | 0 (0.0%) | 2  (10.0%) | 1  (5.0%) | 7.800  (0.454) |
| Depression  (*n*=27) | 15  (55.6%) | 3  (11.1) | 1  (3.7%) | 0 (0.0%) | 3  (11.1%) | 1  (3.7%) | 0 (0.0%) | 2  (7.4%) | 2  (7.4%) | 8.796  (0.283) |
| Hallucination  (*n*=5) | 3  (60.0%) | 0  (0.0%) | 0  (0.0%) | 0 (0.0%) | 0  (0.0%) | 0  (0.0%) | 0 (0.0%) | 2  (40.0%) | 0  (0.0%) | 7.583  (0.569) |

BRV: Brivaracetam

**Table S8** Treatment emergent adverse events in different dose ranges among LEV patients

|  | LEV (*n*=66) | | | | | | | | | |  |
| --- | --- | --- | --- | --- | --- | --- | --- | --- | --- | --- | --- |
|  | **10 ml** | **1000mg** | **1500mg** | **2000mg** | **4ml** | **500mg** | **75mg** | **750mg** | **8 ml** | **Chi-square**  **(*P*-value)** | |
| Aggression  (*n*=37) | 0  (0.0%) | 16  (43.3%) | 4  (10.8%) | 5  (13.5%) | 2  (5.4%) | 4  (10.8%) | 1 (2.7%) | 4  (10.8%) | 1  (2.7%) | 4.896  (0.891) | |
| Headache  (*n*=17) | 0  (0.0%) | 9  (53.0%) | 2  (11.8%) | 2 (11.8%) | 0  (0.0%) | 3  (17.6%) | 1 (5.8%) | 0  (0.0%) | 0  (0.0%) | 6.347  (0.643) | |
| Drowsiness  (*n*=14) | 0  (0.0%) | 7  (50.0%) | 1  (7.2%) | 2 (14.2%) | 0  (0.0%) | 2  (14.2%) | 1 (7.2%) | 1  (7.2%) | 0  (0.0%) | 4.670  (0.858) | |
| Anxiety  (*n*=43) | 1  (2.3%) | 22  (51.1%) | 4  (9.4%) | 6 (13.9%) | 0  (0.0%) | 4  (9.4%) | 1 (2.3%) | 5  (11.6%) | 0  (0.0%) | 9.513  (0.239) | |
| Tension  (*n*=38) | 0  (0.0%) | 19  (50.0%) | 2  (5.2%) | 6 (15.8%) | 0  (0.0%) | 6  (15.8%) | 1 (2.7%) | 3  (7.8%) | 1  (2.7%) | 8.449  (0.341) | |
| Fear  (*n*=19) | 0  (0.0%) | 9  (47.4%) | 3  (15.8%) | 3 (15.8%) | 0  (0.0%) | 1  (5.2%) | 0  (0.0%) | 3  (15.8%) | 0  (0.0%) | 5.406  (0.779) | |
| Insomnia  (*n*=17) | 0  (0.0%) | 6  (35.2%) | 1  (5.9%) | 5 (29.5%) | 0  (0.0%) | 3  (17.6%) | 1 (5.9%) | 1  (5.9%) | 0  (0.0%) | 8.819  (0.312) | |
| Intellectual  (*n*=21) | 0  (0.0%) | 7  (33.4%) | 3  (14.3%) | 2  (9.5%) | 1  (4.7%) | 3  (14.2%) | 0  (0.0%) | 5  (23.9%) | 0  (0.0%) | 10.380  (0.169) | |
| Depression  (*n*=30) | 0  (0.0%) | 17  (56.7%) | 2  (6.7%) | 5  (16.6%) | 0  (0.0%) | 2  (6.7%) | 1  (3.3%) | 3  (10.0%) | 0  (0.0%) | 8.603  (0.321) | |
| Hallucination  (*n*=4) | 0  (0.0%) | 1  (25.0%) | 1  (25.0%) | 1  (25.0%) | 0  (0.0%) | 1  (25.0%) | 0  (0.0%) | 0  (0.0%) | 0  (0.0%) | 7.680  (0.572) | |

LEV: Levetiracetam
